# Comparison of NLP machine learning models with human physicians for ASA Physical Status classification

**DOI:** 10.1038/s41746-024-01259-6

**Published:** 2024-09-28

**Authors:** Soo Bin Yoon, Jipyeong Lee, Hyung-Chul Lee, Chul-Woo Jung, Hyeonhoon Lee

**Affiliations:** 1 Department of Anesthesiology and Pain Medicine, Seoul National University College of Medicine, Seoul National University Hospital, Seoul, Republic of Korea; 2 Department of Anesthesiology and Pain Medicine, Seoul National University Hospital, Seoul, Republic of Korea; 3 Department of Data Science Research, Innovative Medical Technology Research Institute, Seoul National University Hospital, Seoul, Republic of Korea

**Keywords:** Risk factors, Medical research

## Abstract

The American Society of Anesthesiologist’s Physical Status (ASA-PS) classification system assesses comorbidities before sedation and analgesia, but inconsistencies among raters have hindered its objective use. This study aimed to develop natural language processing (NLP) models to classify ASA-PS using pre-anesthesia evaluation summaries, comparing their performance to human physicians. Data from 717,389 surgical cases in a tertiary hospital (October 2004–May 2023) was split into training, tuning, and test datasets. Board-certified anesthesiologists created reference labels for tuning and test datasets. The NLP models, including ClinicalBigBird, BioClinicalBERT, and Generative Pretrained Transformer 4, were validated against anesthesiologists. The ClinicalBigBird model achieved an area under the receiver operating characteristic curve of 0.915. It outperformed board-certified anesthesiologists with a specificity of 0.901 vs. 0.897, precision of 0.732 vs. 0.715, and F1-score of 0.716 vs. 0.713 (all *p* <0.01). This approach will facilitate automatic and objective ASA-PS classification, thereby streamlining the clinical workflow.

## Introduction

The American Society of Anesthesiologists Physical Status (ASA-PS) classification, a fundamental scoring system used to evaluate co-morbidities^1^ and predict perioperative mortality and morbidity^2–5^, is widely used in anesthetic guidelines for non-anesthesia care^6^, ambulatory surgery^7,8^, and pre-procedure evaluations^9^. This has enabled anesthesiologists to provide patients with the benefits of sedation or analgesia while minimizing the associated risks. The ASA-PS classification has significantly impacted the healthcare system, particularly on billing and reimbursement by health insurance companies^10,11^. However, poor to moderate agreement has been observed for the use of the ASA-PS system among healthcare professionals across various departments and patient groups, leading to inconsistencies^12–14^, which have hindered its objective use. Moreover, significant discrepancies persist in different patient scenarios despite a 2014 update providing approved examples for each ASA-PS class^11,15^. Thus, the development of a reliable tool capable of assigning ASA-PS classes accurately by extracting meaningful data from unstructured patient information is necessary.

Recent advances in the field of natural language processing (NLP) have led to significant improvement in the management of unstructured medical text data. For instance, Generative Pretrained Transformer (GPT)-4 (OpenAI, San Francisco, California, USA) has demonstrated exceptional accuracy of >90% on the United States Medical Licensing Examination (USMLE). However, its lower performance on more specialized tasks indicates the requirement for targeted improvements^16,17^. BioClinicalBERT, trained on a large domain-specific biomedical text corpus, has exhibited promising results in ASA-PS classification, with a macro-average area under the receiver operating characteristic curve (AUROC) of 0.845. Furthermore, its processing limit of 512 tokens indicates potential areas for improvement^18^. ClinicalBigBird, which can model up to 4,096 tokens, may leverage long-term dependencies, as demonstrated in clinical question answering and medical document classification^19^. These specialized NLP models can also enhance patient privacy and mitigate misclassification errors by effectively utilizing their extensive token capacity and domain-specific knowledge, thus suggesting their potential to outperform general models in medical tasks.

This study aims to develop an NLP-based ASA-PS classification model that uses free-text pre-anesthesia evaluation summaries and compare its performance with that of different levels of human physicians, including anesthesiology residents and board-certified anesthesiologists. The integration of cutting-edge NLP algorithms into the ASA-PS classification could lead to the creation of an automatic and objective framework for risk prediction, shared decision-making, and resource allocation in perioperative medicine.

## Results

### Data construction

**Fig. 1:**
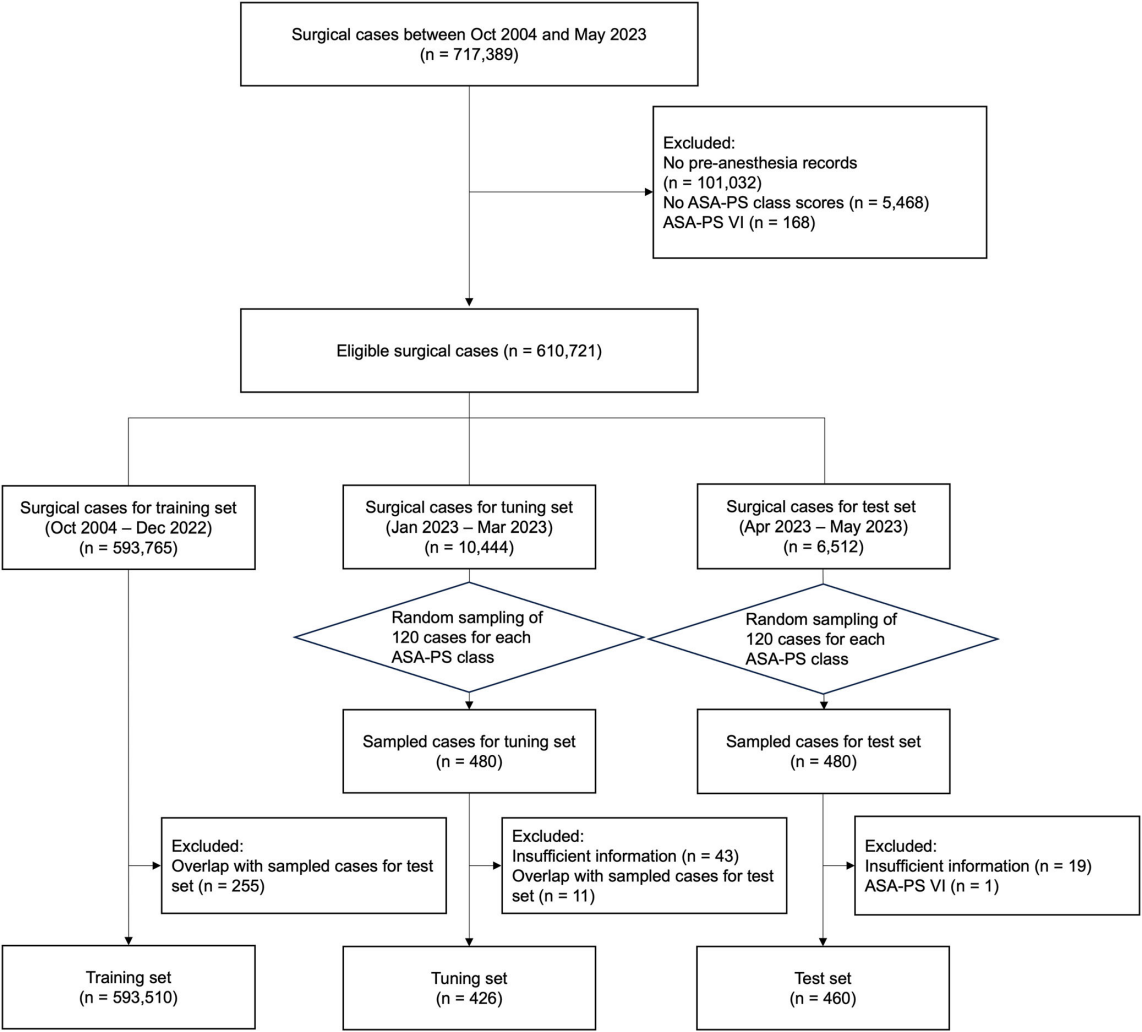
Flowchart of study design. The flowchart illustrates the study design for developing and evaluating the natural language processing-based ASA-PS classification models. The study utilized data from 717,389 surgical cases recorded at a tertiary academic hospital between October 2004 and May 2023. The data was divided into three distinct datasets: training, tuning, and test datasets. ASA-PS, American Society of Anesthesiologists Physical Status.

The training (*n* = 593,510), tuning (*n* = 426), and test (*n* = 460) sets comprised patients (*n* = 717,389) who underwent surgery between October 2004 and May 2023 (Fig. 1). Table 1 presents the characteristics of the datasets. ASA-PS scores of I or II were assigned to 90% of the training dataset. In contrast, the distribution of classes in the tuning and test datasets was relatively uniform, ensuring equal representation of each ASA-PS class to fairly assess the rare classes assigned as III or IV–V. An increase in the length of pre-anesthesia evaluation summaries with higher ASA-PS score classifications was also observed. A verification process involving the application of regular expressions and subsequent manual review confirmed that no residual ASA-PS information remained in the pre-anesthesia evaluation summaries. The racial composition of our study cohort remained predominantly Asian at over 99.5%, with White non-Hispanic patients comprising 0.3%, Black or African American patients 0.1%, and others 0.1%.

**Table 1:** Dataset characteristics

Characteristics	Training set	Tuning set	Test set
**Period**	Oct 2004 to Dec 2022	Jan 2023 to Mar 2023	Apr 2023 to May 2023
**Number of cases**	593,510	426	460
**Age, mean (SD)**	43.7 (25.2)	57.5 (17.4)	56.1 (16.7)
**Female, no. (%)**	312,921 (52.7%)	209 (49.0%)	281 (61.0%)
**Anesthesia type, no. (%)**			
General	487,801 (82.19%)	345 (80.99%)	379 (82.39%)
Regional	53,618 (9.04%)	44 (10.32%)	51 (11.09%)
MAC	52,091 (8.77%)	37 (8.69%)	30 (6.52%)
**ASA-PS classification, no. (%)**			
I	244,386 (41.18%)	65 (15.26%)	75 (16.30%)
II	291,349 (49.09%)	128 (30.05%)	171 (37.17%)
III	54,092 (9.11%)	147 (34.50%)	163 (35.43%)
IV-V	3683 (0.62%)	86 (20.19%)	51 (11.09%)
**Number of tokens and words in pre-anesthesia evaluation, median (IQR)**			
**Token count**	68 (27, 139)	232 (82, 440)	190 (110, 321)
ASA-PS I	29 (10, 60)	41 (26, 61)	64 (46, 114)
ASA-PS II	97 (53, 161)	105 (60, 168)	131 (101, 187)
ASA-PS III	238 (140, 398)	338 (233, 454)	286 (215, 406)
ASA-PS IV-V	317 (153, 565)	654 (423, 1136)	679 (370, 1184)
**Word count**	163 (63, 352)	532 (186, 1131)	462 (257, 818)
ASA-PS I	70 (24, 150)	99 (60, 140)	145 (94, 283)
ASA-PS II	230 (121, 406)	240 (136, 414)	313 (222, 462)
ASA-PS III	642 (356, 1158)	812 (581, 1209)	736 (532, 994)
ASA-PS IV-V	889 (395, 1726)	1680 (1052, 3053)	1677 (974, 3325)

*MAC* monitored anesthesia care, *ASA-PS* American Society of Anesthesiologists Physical Status.

### Performance of the NLP models

**Fig. 2:**
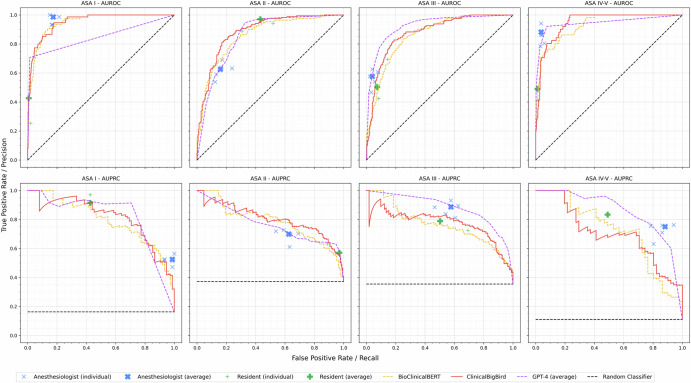
Receiver operating characteristic and precision-recall curves of the models and human physicians for each ASA-PS class. The curves of the ClinicalBigBird model were compared with those of BioClinicalBERT, GPT-4, anesthesiology residents, and board-certified anesthesiologists. AUROC area under the receiver operating characteristic curve, AUPRC area under the precision-recall curvem, ASA-PS American Society of Anesthesiologists Physical Status, GPT-4 Generative Pretrained Transformer-4.

The performance of the NLP models, anesthesiology residents, and board-certified anesthesiologists was compared with the consensus reference labels of ASA-PS scores assigned by the board-certified anesthesiologist consensus committee by calculating the AUROC. Figure 2 and Table 2 present the performances of individual ASA-PS raters and the average values for the board-certified anesthesiologists and anesthesiology residents. Supplementary Table 3 details the ASA-PS classification of each board-certified anesthesiologist and anesthesiology resident. The Fleiss’ kappa values for the board-certified anesthesiologists, anesthesiology residents, and ten different responses of GPT-4 were 0.743 (95% confidence interval [CI] 0.731-0.754), 0.480 (95% CI 0.463-0.498), and 0.612 (95% CI 0.601-0.623), respectively (Supplementary Table 4).

**Table 2:** ASA-PS Classification performances of the NLP models, anesthesiology residents, and board-certified anesthesiologists

	AUROC	AUPRC	Sensitivity	Specificity	Precision	F1-score
**ClinicalBigBird (our model)**						
Weighted-average	0.912 (0.905–0.918)	0.804 (0.786–0.819)	0.767 (0.750–0.783)	0.870 (0.862–0.878)	0.762 (0.745–0.777)	0.754 (0.737–0.769)
Macro-average	0.915 (0.909–0.920)	0.774 (0.758–0.789)	0.723 (0.708–0.737)	0.901 (0.897–0.906)	0.732 (0.717–0.746)	0.716 (0.701–0.730)
Micro-average	0.914 (0.909–0.919)	0.745 (0.728–0.761)	0.728 (0.715–0.741)	0.728 (0.715–0.741)	0.728 (0.715–0.741)	0.728 (0.715–0.741)
ASA-PS I	0.952 (0.945–0.958)	0.814 (0.783–0.840)	0.680 (0.645–0.716)	0.974 (0.969–0.980)	0.836 (0.804–0.866)	0.750 (0.721–0.776)
ASA-PS II	0.884 (0.874–0.894)	0.803 (0.782–0.823)	0.871 (0.854–0.889)	0.772 (0.755–0.788)	0.693 (0.672–0.714)	0.772 (0.755–0.787)
ASA-PS III	0.877 (0.866–0.888)	0.775 (0.752–0.799)	0.595 (0.570–0.620)	0.926 (0.916–0.936)	0.815 (0.791–0.837)	0.688 (0.667–0.708)
ASA-PS IV-V	0.946 (0.938–0.954)	0.704 (0.665–0.743)	0.745 (0.702–0.786)	0.934 (0.926–0.942)	0.584 (0.541–0.623)	0.654 (0.619–0.689)
**BioClinicalBERT**						
Weighted-average	0.897 (0.889–0.904)	0.787* (0.771–0.802)	0.657* (0.637–0.675)	0.883* (0.876–0.891)	0.724* (0.705–0.743)	0.685* (0.667–0.704)
Macro-average	0.899 (0.893–0.906)	0.766* (0.751–0.783)	0.679* (0.662–0.694)	0.886* (0.880–0.890)	0.666* (0.650–0.680)	0.661* (0.645–0.677)
Micro-average	0.901 (0.895–0.907)	0.739* (0.724–0.755)	0.676* (0.661–0.690)	0.676* (0.661–0.690)	0.676* (0.661–0.690)	0.676* (0.661–0.690)
ASA-PS I	0.947 (0.940–0.954)	0.796* (0.766–0.822)	0.587* (0.550–0.623)	0.964* (0.957–0.970)	0.759* (0.721–0.796)	0.662* (0.630–0.692)
ASA-PS II	0.860 (0.849–0.872)	0.786* (0.765–0.806)	0.713* (0.690–0.737)	0.820* (0.806–0.835)	0.701* (0.678–0.725)	0.707* (0.688-0.726)
ASA-PS III	0.860 (0.849–0.872)	0.764* (0.743–0.784)	0.650* (0.628–0.673)	0.858* (0.846-0.870)	0.716* (0.691–0.738)	0.682* (0.663–0.701)
ASA-PS IV-V	0.929 (0.918–0.940)	0.720* (0.681–0.758)	0.764* (0.726–0.804)	0.900* (0.890–0.909)	0.486* (0.449–0.525)	0.594* (0.561–0.628)
**GPT-4 (average)**						
Weighted-average	0.859 (0.850–0.869)	0.722* (0.702–0.742)	0.559* (0.542–0.575)	0.859* (0.850–0.867)	0.759* (0.736–0.780)	0.576* (0.555–0.597)
Macro-average	0.893 (0.885–0.900)	0.769* (0.753–0.784)	0.594* (0.578–0.610)	0.881* (0.876–0.886)	0.799* (0.784–0.814)	0.623* (0.605–0.639)
Micro-average	0.899 (0.893–0.906)	0.776 (0.762–0.790)	0.695* (0.681–0.710)	0.695* (0.681–0.710)	0.695* (0.681–0.710)	0.695* (0.681–0.710)
ASA-PS I	0.847* (0.830–0.864)	0.701* (0.669–0.734)	0.239* (0.207–0.270)	0.995* (0.992–0.997)	0.899* (0.850–0.942)	0.377* (0.335–0.417)
ASA-PS II	0.856 (0.845–0.867)	0.713* (0.690–0.738)	0.766* (0.744–0.787)	0.757* (0.741–0.774)	0.651* (0.628–0.673)	0.704* (0.685–0.722)
ASA-PS III	0.924† (0.915–0.932)	0.850* (0.833–0.867)	0.902* (0.887–0.917)	0.774* (0.758–0.790)	0.687* (0.665–0.709)	0.780* (0.764–0.796)
ASA-PS IV-V	0.945 (0.931–0.959)	0.810* (0.778–0.842)	0.470* (0.422–0.514)	0.998* (0.996–0.999)	0.960* (0.933–0.983)	0.630* (0.583–0.671)
**Board-certified anesthesiologist (average)**						
Weighted-average	NA		0.772* (0.760–0.785)	0.847* (0.838–0.855)	0.645* (0.629–0.662)	0.676* (0.659–0.692)
Macro-average			0.768* (0.756–0.779)	0.897* (0.893–0.902)	0.715* (0.702–0.729)	0.713* (0.700–0.727)
Micro-average			0.696* (0.682–0.709)	0.696 (0.682–0.709)	0.696† (0.682–0.709)	0.696 (0.682–0.709)
ASA-PS I			0.987* (0.977–0.995)	0.826* (0.813–0.839)	0.525* (0.498–0.555)	0.686* (0.662–0.711)
ASA-PS II			0.626* (0.602–0.650)	0.841* (0.827–0.854)	0.699* (0.675–0.723)	0.660* (0.641–0.681)
ASA-PS III			0.577* (0.551–0.602)	0.960* (0.952–0.967)	0.887* (0.867–0.907)	0.699* (0.677–0.719)
ASA-PS IV-V			0.882* (0.849–0.910)	0.963* (0.957–0.969)	0.748* (0.711–0.787)	0.809* (0.783–0.836)
**Anesthesiology resident (average)**						
Weighted-average	NA		0.701* (0.685–0.717)	0.778* (0.769–0.788)	0.734* (0.716–0.751)	0.652* (0.632–0.671)
Macro-average			0.598* (0.583–0.614)	0.868* (0.864–0.873)	0.777* (0.760–0.794)	0.633* (0.616–0.650)
Micro-average			0.663* (0.648–0.678)	0.663gjgjgjgjgj (0.648–0.678)	0.663* (0.648–0.678)	0.663* (0.648–0.678)
ASA-PS I			0.427* (0.389–0.463)	0.992* (0.989–0.995)	0.915* (0.882–0.946)	0.582* (0.544–0.617)
ASA-PS II			0.971* (0.962–0.980)	0.568* (0.549–0.587)	0.570* (0.552–0.590)	0.719* (0.703–0.735)
ASA-PS III			0.503* (0.478–0.529)	0.926 (0.917-0.936)	0.789* (0.762-0.816)	0.614* (0.591–0.637)
ASA-PS IV-V			0.491* (0.446–0.537)	0.988* (0.984–0.991)	0.832* (0.787–0.878)	0.617* (0.576–0.658)

Data are presented as means with 95% confidence intervals. The DeLong test and Mann–Whitney *U*-test were conducted to compare the AUROC and AUPRC of two different models, respectively. Statistical significance is indicated by *, † for *p* <0.001 and *p* <0.01, respectively, in comparison with the performance of ClinicalBigBird. *ASA-PS* American Society of Anesthesiologists Physical Status, *NLP* natural language processing, *AUROC* area under the receiver operating characteristic curve, *AUPRC* area under the precision-recall curve, *NA* not applicalble.

The ClinicalBigBird-based ASA-PS classification model achieved AUROCs of >0.91 in both macro- and micro-averages (Table 2). The ClinicalBigBird outperformed the BioClinicalBERT in micro-averaged AUROC (*p* = 0.010) and was comparable to GPT-4 in macro- and micro-averaged AUROCs for ASA-PS classification. Both the aleatoric and epistemic uncertainties of the ClinicalBigBird were lower than those of the BioClinicalBERT (Supplementary Table 5). Subgroup analysis revealed that the ClinicalBigBird achieved greater AUROCs and area under the precision-recall curves (AUPRCs) for inputs longer than the median length of pre-anesthesia evaluation summaries compared to shorter ones (Table 3). In particular, for longer text inputs, the ClinicalBigBird significantly outperformed GPT-4 in ASA-PS I and II, and also outperformed BioClinicalBERT in ASA-PS classes II to IV–V.

**Table 3:** Performance of NLP-based models in ASA-PS classification across subgroups, stratified according to the length of pre-anesthesia evaluation summaries, with the median length of dataset as the threshold

	≤Median length (*N* = 225)		>Median length (*N* = 235)	
	AUROC	AUPRC	AUROC	AUPRC
ClinicalBigBird				
Weighted-average	0.908 (0.901–0.915)	0.820 (0.805–0.834)	0.931 (0.926–0.936)	0.853 (0.842–0.865)
Macro-average	0.919 (0.913–0.924)	0.785 (0.770–0.800)	0.926 (0.921–0.931)	0.814 (0.799–0.827)
Micro-average	0.912 (0.906–0.917)	0.753 (0.737–0.769)	0.918 (0.912–0.923)	0.743 (0.725–0.759)
ASA-PS I	0.979 (0.975–0.983)	0.891 (0.867–0.914)	0.957 (0.951–0.963)	0.852 (0.830–0.872)
ASA-PS II	0.848 (0.836–0.861)	0.771 (0.750–0.791)	0.921 (0.913–0.929)	0.867 (0.851–0.881)
ASA-PS III	0.900 (0.890–0.909)	0.774 (0.746–0.798)	0.863 (0.851–0.875)	0.799 (0.778–0.820)
ASA-PS IV-V	0.947 (0.939–0.953)	0.703 (0.664–0.739)	0.963 (0.958–0.969)	0.736 (0.698–0.773)
BioClinicalBERT				
Weighted-average	0.897 (0.889–0.905)	0.807* (0.791–0.820)	0.907 (0.900–0.913)	0.830* (0.818–0.841)
Macro-average	0.902 (0.895–0.909)	0.780* (0.764–0.796)	0.903 (0.897–0.909)	0.796* (0.782–0.808)
Micro-average	0.905 (0.899–0.911)	0.757* (0.742–0.772)	0.896† (0.890–0.902)	0.722* (0.705–0.737)
ASA-PS I	0.957 (0.951–0.963)	0.838* (0.812–0.861)	0.964 (0.957–0.970)	0.894* (0.874–0.911)
ASA-PS II	0.845 (0.832–0.858)	0.782* (0.760–0.801)	0.874† (0.864–0.885)	0.796* (0.776–0.814)
ASA-PS III	0.906 (0.897–0.915)	0.814* (0.792–0.836)	0.819 (0.806–0.832)	0.733* (0.712–0.755)
ASA-PS IV-V	0.901 (0.886–0.914)	0.688* (0.648–0.727)	0.956 (0.949–0.962)	0.760* (0.722–0.793)
GPT-4 (average)				
Weighted-average	0.910 (0.901–0.918)	0.800* (0.780–0.818)	0.816† (0.805–0.827)	0.656* (0.636–0.675)
Macro-average	0.914 (0.907–0.921)	0.820* (0.806–0.834)	0.877 (0.871–0.884)	0.733* (0.718–0.747)
Micro-average	0.919 (0.913–0.925)	0.820* (0.807-0.832)	0.879† (0.871–0.884)	0.732* (0.717-0.747)
ASA-PS I	0.908 (0.893-0.922)	0.784* (0.753-0.815)	0.790* (0.770–0.809)	0.647* (0.614-0.680)
ASA-PS II	0.909 (0.900–0.917)	0.801* (0.780–0.821)	0.813* (0.801–0.827)	0.624* (0.600–0.651)
ASA-PS III	0.922 (0.913–0.931)	0.861* (0.845–0.877)	0.931† (0.923–0.938)	0.847* (0.829–0.865)
ASA-PS IV-V	0.917 (0.899–0.933)	0.835* (0.804–0.864)	0.975 (0.971–0.979)	0.813* (0.783–0.842)

Data are presented as mean with a 95% confidence interval. Statistical significance is indicated by *, † for *p* <0.001 and *p* <0.01, respectively. *AUROC* area under the receiver operating characteristic curve, *AUPRC* area under the precision-recall curve, *ASA-PS* American Society of Anesthesiologists Physical Status.

### Human physicians *vs*. NLP model

Performance of the ClinicalBigBird model was higher than board-certified anesthesiologists with specificity 0.901 vs. 0.897, precision 0.732 vs. 0.715, and F1-score 0.716 vs. 0.713, respectively, all *p* <0.01. Moreover, the ClinicalBigBird model outperformed the anesthesiology residents in sensitivity 0.723 vs. 0.598, specificity 0.901 vs. 0.868, and F1-score 0.716 vs. 0.633, respectively, all *p* <0.001.

**Fig. 3:**
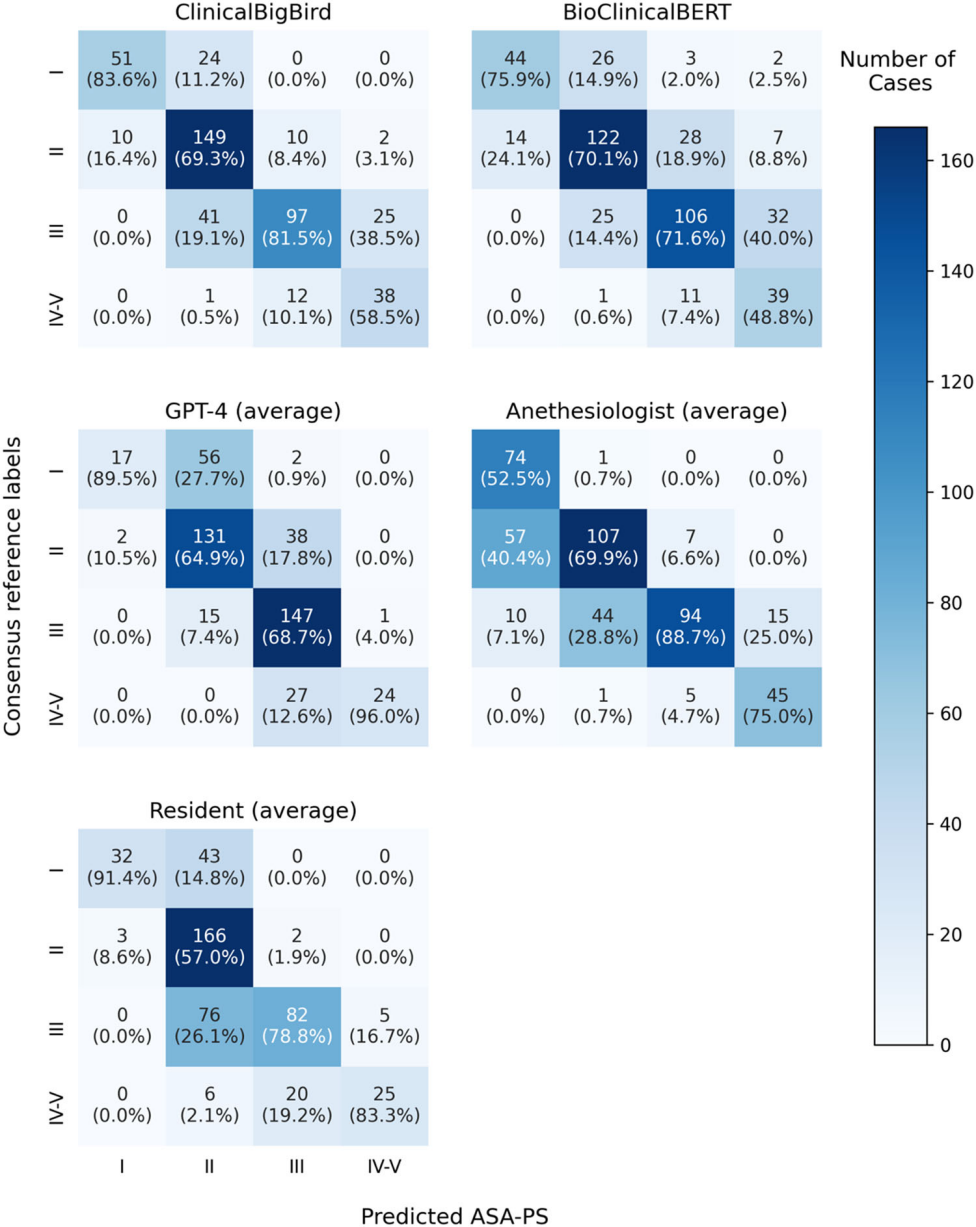
Confusion matrices of the natural language processing models (ClinicalBigBird, BioClinicalBERT, and GPT-4) and human physicians (anesthesiology residents and board-certified anesthesiologists) for ASA-PS classification. Each cell presents the number of case and the corresponding percentages that represent the proportion of cases correctly classified by each model or physician group within each predicted ASA-PS class. ASA-PS American Society of Anesthesiologists Physical Status, GPT-4 Generative Pretrained Transformer-4.

Evaluation of the confusion matrices (Fig. 3) revealed that the anesthesiology residents frequently classified over half of the pre-anesthesia records (63.26%) as ASA-PS II. In contrast, the board-certified anesthesiologists often underestimated these classifications and misidentified ASA-PS II as ASA-PS I and ASA-PS III as ASA-PS I or II at rates of 33.33% and 33.13%, respectively. The ClinicalBigBird demonstrated improved performance in these categories. The underestimation rates for ASA-PS II and ASA-PS III were 5.85% and 25.15%, respectively. However, an increase in the overestimation rate for ASA-PS I from 1.35% to 32.00% partially offset this improvement. GPT-4 exhibited a significant tendency toward overestimation with rates of 77.33% and 22.22% for ASA-PS I and ASA-PS II, respectively. These rates were higher than those observed in the other models and all physician groups.

### Feature importance

**Fig. 4:**
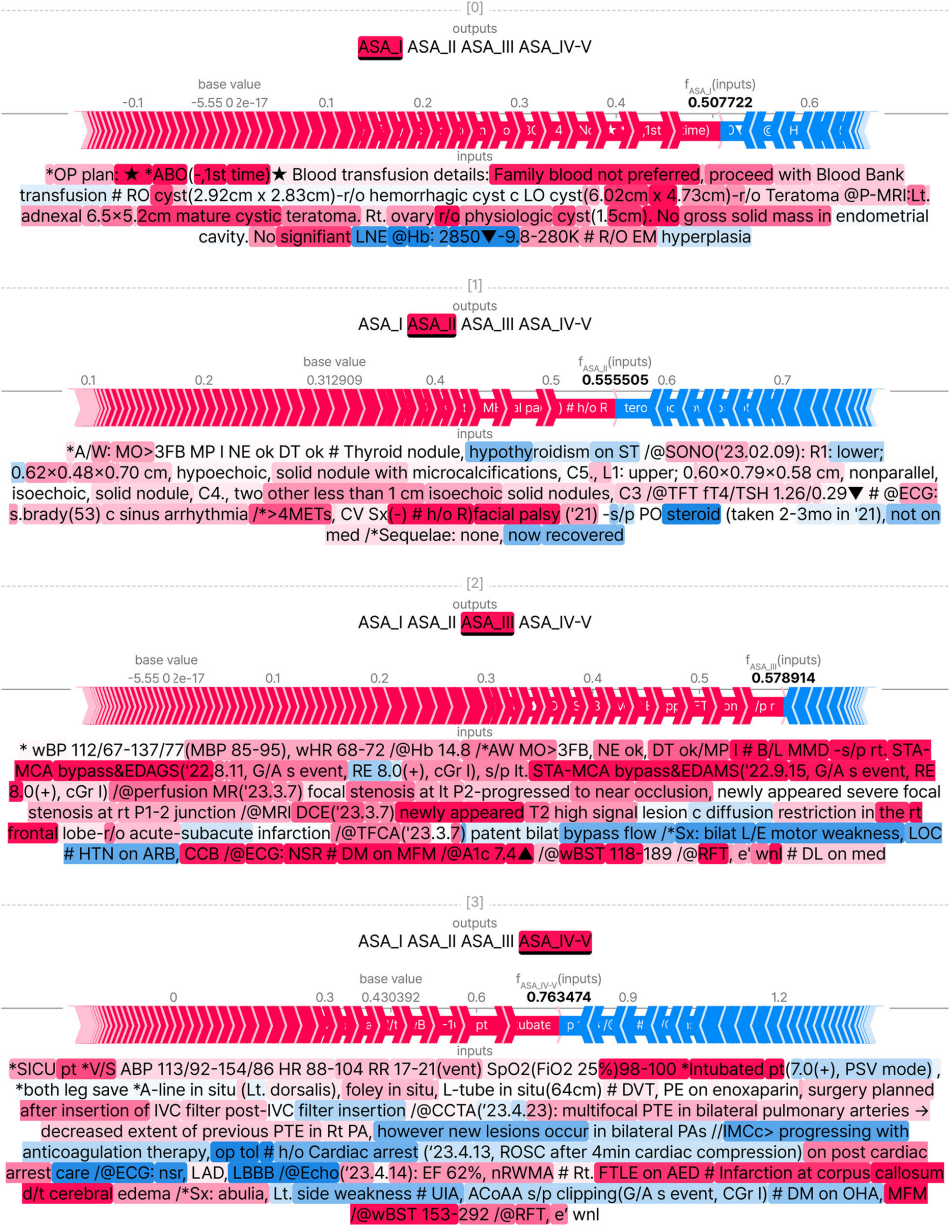
Shapley plot demonstrating the effect of input texts on each ASA-PS classification in the ClinicalBigBird model. Each force plot shows the contribution of input features (tokens) to the output probability of a specific ASA-PS class. The base value represents the average model output, and the output value is the model’s prediction for the given instance. Tokens in red contribute positively towards pushing the model output from the base value to the predicted value (indicating a higher probability of the class), while tokens in blue contribute negatively (indicating a lower probability of the class). The magnitude of the contribution is shown by the size of the arrows. This visualization helps to understand which features (tokens) are driving the model’s predictions and their respective contributions to the final Shapley score. ASA-PS American Society of Anesthesiologists Physical Status, ABO ABO blood group system, r/o rule out, RO right ovary, LO left ovary, P-MRI pelvic magnetic resonance imaging, LNE lymph node enlargement, EM endometrial, A/W or AW airway, MP mallampati, NE neck extension, DT Interincisor Gap, ST synthroid, SONO sonogram, TFT thyroid function test, fT4 free T4, TSH thyroid stimulating hormone, ECG electrocardiogram, s.brady sinus bradycardia, c with, METs metabolic equivalents, CV cardiovascular, Sx symptoms, h/o history of, s/p status post, PO per os, med medication, wBP ward blood pressure, MBP mean blood pressure, wHR ward heart rate, B/L bilateral, MMD moyamoya disease, STA-MCA superficial temporal artery to middle cerebral artery, EDAGS encephalo-duro-arterio-galeo-synangiosis, G/A general anesthesia, RE reinforced tube, cGr cormack grade, perfusion MR perfusion magnetic resonance, MRI DCE magnetic resonance imaging dynamic contrast enhanced, TFCA transfemoral cerebral angiogram, L/E lower extremities, LOC loss of consciousness, HTN hypertension, ARB angiotensin receptor blocker, CCB calcium channel blocker, NSR normal sinus rhythm, A1c hemoglobin A1c, wBST ward blood sugar test, RFT renal function test, e’ electrolyte, DL dyslipidemia, SICU surgical intensive care unit, pt patient, V/S vital signs, ABP arterial blood pressure, HR heart rate, RR respiratory rate, vent ventilator, SpO2 peripheral oxygen saturation, FiO2 fraction of inspired oxygen, PSV pressure support ventilation, A-line arterial line, Lt left, foley foley catheter, L-tube levin tube, DVT deep vein thrombosis, PE pulmonary embolism, IVC inferior vena cava, CCTA coronary computed tomography angiography, PTE pulmonary thromboembolism, PA pulmonary artery, IMCc internal medicine cardiology consult; op tol, operative tolerance, ROSC return of spontaneous circulation, LAD left anterior descending, LBBB left bundle branch block, Echo echocardiogram, EF ejection fraction, nRWMA no regional wall motion abnormalities, FTLE focal to bilateral tonic-clonic seizure, AED antiepileptic drug, UIA unruptured intracranial aneurysm, ACoAA anterior communicating artery aneurysm, DM diabetes mellitus, OHA oral hypoglycemic agent, MFM metformin, wnl within normal limits.

Figure 4 illustrates how a specific input text contributes to the prediction performance of the model for each ASA-PS class. The Shapley values of a few key input texts, such as hypothyroidism, moyamoya disease, and infarction, used to classify the ASA-PS according to the ASA-PS guidelines were higher than those of other texts.

## Discussion

An NLP-based ASA-PS classification model was developed in this study using unstructured pre-anesthesia evaluation summaries. This model exhibited a performance comparable with that of board-certified anesthesiologists in the ASA-PS classification. Furthermore, the model outperformed other NLP-based models, such as BioClinicalBERT and GPT-4.

This study is the first to compare the performance of NLP models with that of trained physicians with various levels of expertise in a domain-specialized task. The ClinicalBigBird-based ASA-PS classification model showed higher specificity, precision, and F1-score than that of the board-certified anesthesiologists. These findings indicate that the NLP-based approach can automatically and consistently assign ASA-PS classes using the pre-anesthesia evaluation summaries in streamlined clinical workflows with an accuracy similar to that of anesthesiologists.

The low inter-rater reliability of the ASA-PS classification remains a long-standing issue in clinical and healthcare settings. The 2014 ASA-PS classification guidelines introduced additional examples; however, these examples could not fully resolve the uncertainty as they do not cover all comorbidities. Physicians assigned the correct ASA-PS classifications in 7 out of 10 cases after the addition of examples; nevertheless, they failed to reach a consensus in one-third of the cases^20^. This study found moderate agreement among anesthesiologists (Fleiss’ kappa value, 0.743), consistent with previous research reporting weighted kappa values ranging from 0.62 to 0.86^21^. The algorithmic approach could address the variability in the inter-rater discrepancies by providing a unified, data-driven score per scenario based on consensus reference labels.

Differentiating ASA-PS II from ASA-PS III is particularly important in clinical decision-making^20^. Several guidelines^7,9^ and regulations^6,8,14^ state that differentiating ASA-PS II from ASA-PS III plays a critical role in formulating a plan for non-anesthesia care and ambulatory surgery. Patients classified as ASA-PS III or higher often require additional evaluation before surgery. Errors in assignment can lead to the over- or underprescription of preoperative testing, thereby compromising patient safety^22^. The board-certified anesthesiologists and the anesthesiology residents exhibited error rates of 13.48% and 21.96%, respectively, in assigning ASA-PS I or II as III or IV–V, or vice versa. However, the ClinicalBigBird developed in this study demonstrated a lower error rate of 11.74%, outperforming the error rates of physicians and other NLP-based models, such as BioClinicalBERT (14.12%) and GPT-4 (11.95%).

The ClinicalBigBird model frequently misclassified ASA-PS III cases as ASA-PS IV-V, while the anesthesiology residents misclassified ASA-PS IV-V cases as ASA-PS III, resulting in low sensitivity (Fig. 3). This discrepancy may arise because the board-certified anesthesiologists providing intraoperative care rate the patient as having higher severity, whereas residents classify the same patient as having lower severity^23,24^. Our model, ClinicalBigBird, was fine-tuned with consensus reference labels, thereby rating ASA-PS III cases as having higher severity, mimicking the board-certified anesthesiologists. Furthermore, anesthesiology residents tended to rate conservatively toward ASA-PS II, possibly due to limited clinical experience^25^. Conversely, the board-certified anesthesiologists often misclassified ASA-PS II cases as ASA-PS I, which might be caused by overlooking well-controlled comorbidities. However, the NLP models, particularly ClinicalBigBird, can systemically process all available information without fatigue or bias. This capacity potentially mitigates the risk of overlooking pertinent clinical details and facilitates a balanced assessment.

The fine-tuned ClinicalBigBird model was superior to GPT-4 in terms of performance. The performance of GPT-3.5 was comparable to that of board-certified anesthesiologists in six out of ten hypothetical scenarios, although it tended to underestimate ASA-PS IV-V^25^. However, GPT-4 tended to overestimate the ASA-PS scores in the present study. GPT-4 often misclassified ASA-PS I and ASA-PS II as ASA-PS III in the confusion matrix owing to false inferences regarding underlying diseases and systemic conditions. It is important to note that GPT-4 was utilized through general prompting rather than task-specific fine-tuning in this study, unlike BioClinicalBERT and ClinicalBigBird which were optimized for ASA-PS classification. While the findings of the present study suggest that GPT-4 is currently less optimal for ASA-PS classification than other language models, this comparison has limitations. If GPT-4 were to undergo domain-specific pretraining and task-specific fine-tuning similar to the other models, its performance could potentially improve significantly^26^, possibly even surpassing the current top-performing models.

The performance of several NLP models for assigning the ASA-PS classes was compared in the present study. BioClinicalBERT, which is limited to an input sequence of 512 tokens, requires truncation and segmentation of the input text. It achieved a macro-average AUROC of 0.899, slightly higher than the previous study, which achieved a macro-average AUROC of 0.845^18^. In addition, the study investigated the fastText model, which can process all texts in clinical notes and achieved a macro-average AUROC of 0.865. However, since the fastText model was not pretrained with a medical text corpus, its performance might still be limited. On the other hand, the ClinicalBigBird, used in our study, has two advantages; it is pre-trained with a large medical corpus and capable of processing up to 4096 tokens^19^. Therefore, the ClinicalBigBird-based model is well-suited for processing long, unstructured text, thereby streamlining clinical workflows and offering advantages over machine learning models that require structured data inputs such as words, numbers, or binaries^27,28^.

A key limitation of our study is the inherent subjectivity in ASA-PS classification. Despite our rigorous consensus-based approach yielding a good agreement (Fleiss’ kappa of 0.743) compared to previous studies (Fleiss’ kappa of 0.48–0.69) ^12,27^, some uncertainty in the ground truth persists, suggesting our performance metrics should be interpreted as approximate rather than definitive. While this improvement is noteworthy, it’s important to recognize that perfect agreement in ASA-PS classification remains challenging due to its subjective nature. Future research in this area should continue to focus on methods to enhance inter-rater reliability while acknowledging the balance between achievable agreement and the inherent variability in clinical assessments^29^.

This study also had other limitations. First, the ClinicalBigBird and BioClinicalBERT models were developed and validated using pre-anesthesia evaluation summaries from a single institution in South Korea. Future studies should focus on validating these models’ performance owing to the differences in patient demographics such as nationality, race, and ethnicity, and writing styles of the pre-anesthesia evaluation summaries. In addition, the present study only included adult patients owing to the limitations of the ASA-PS classification systems in pediatric cases. However, the success of the machine learning algorithm in classifying the ASA-PS scores in pediatric patients suggests that a more comprehensive ASA-PS classification model can be constructed using the NLP approach. Second, the small sample size of five board-certified anesthesiologists and three anesthesiology residents may not be representative of the broader population of these professionals. A more rigorous and generalizable conclusion would require a larger and more diverse sample size, encompassing data from different hospitals in various regions or countries. This would facilitate a more comprehensive analysis of the variability of the ASA-PS classification across different clinical settings. Third, only free-text pre-anesthesia evaluation summaries refined by physicians were used in this study. Applying the NLP technique to unprocessed medical records, such as outpatient history, nursing notes, admission intake, and laboratory values, would result in a broader scope for generalization and a more significant impact on clinical practice. Fourth, translating the pre-anesthesia evaluation texts from Korean to English may have affected the accuracy of the ASA-PS classification model. Fifth, the use of static few-shot prompting for GPT-4 ensured consistency across predictions but may limit the model’s ability to adapt to a broader range of clinical scenarios not represented in the demonstrations. Future research could explore the impact of dynamic few-shot prompting for GPT-4 to enhance the model’s robustness or generalizability across diverse clinical cases. Sixth, comparing GPT-4’s performance directly with models like BioClinicalBERT and ClinicalBigBird is limited by the fact that GPT-4 was only prompted and not fine-tuned on task-specific data, which could potentially affect its performance outcome. Finally, prevalence-dependent metrics such as the F1-score may not fully represent model performance in diverse clinical settings due to differences in ASA-PS class distributions between our tuning and test sets compared to the general population.

In conclusion, an NLP-based model for the ASA-PS classification using free-text pre-anesthesia evaluation summaries as input can achieve a performance similar to that of board-certified anesthesiologists. This approach can improve the consistency and inter-rater reliability of the ASA-PS classification in healthcare systems if confirmed in clinical settings.

## Methods

### Study design

This observational study was approved by the Institutional Review Board (IRB) of the Ethics Committee of Seoul National University Hospital (approval number: 2306-167-1444). The IRB waived the requirement for obtaining informed consent from the patients owing to the retrospective nature of this study. The study adhered to the Standards for Reporting of Diagnostic Accuracy Studies (STARD) and other relevant guidelines^30^.

### Data collection

Patients who underwent surgical procedures at the Seoul National University Hospital between October 2004 and May 2023 were eligible for inclusion in this study. The exclusion criteria were as follows: absence of pre-anesthesia evaluation summaries or ASA-PS scores, brain death, and ASA-PS class VI (because of the direct correspondence between the diagnosis and the ASA-PS score).

Data regarding pre-anesthesia evaluations and the physician-assigned ASA-PS scores were extracted from the Seoul National University Hospital Patient Research Environment (SUPREME) system. In addition, data regarding patient demographics, medical history, surgical history, laboratory test results, diagnosis, and medications were also extracted. ASA-PS classes IV and V were merged into the “IV–V” class to balance the rare classes in the dataset^18,20^. In addition, the modifier “E” for emergency surgery was also removed. Thus, the final ASA-PS classification system comprised the following classes: I, II, III, and IV–V.

The datasets for training, tuning, and testing were initially segmented based on the periods during which patients underwent surgical procedures from October 2004–December 2022, January 2023–March 2023, and April 2023–May 2023, respectively. For the tuning and test sets, to ensure a feasible scope for the intensive manual labeling process by a consensus committee of five board-certified anesthesiologists, we randomly subsampled 120 patients aged >18 years from each ASA-PS class from the larger pools of the datasets. This stratified random sampling approach was designed to ensure a balanced representation of all the ASA-PS classes in our evaluations, including classes III and IV–V, to prevent bias from the underrepresentation of these rarer classes and ensure a fair assessment across all ASA-PS classes. Cases were also excluded during the labeling process if board-certified anesthesiologists determined that the pre-anesthesia evaluation summaries contained insufficient information. Lastly, to maintain the integrity of the test set, any patients in training and tuning sets who also had surgical records in the test set were removed from the training and tuning sets, ensuring that the datasets comprised completely disjointed sets of patients with separate identifiers.

### Data preparation

Data preparation was performed initially using the pre-anesthesia evaluation summaries. A proprietary translator was employed to translate the summaries written in a mixture of Korean and English into English across all datasets. Supplementary Table 1 presents a sample translation. The byte-pair encoding technique was used to segment the sentences in the evaluations into tokens^31^. To ensure the ASA-PS classification was not documented in the pre-anesthesia evaluation summaries, we used regular expressions to detect and remove any explicit mentions of ASA classifications within the summaries. This process was further verified by manually reviewing the tuning and test sets to confirm no residual ASA-PS information remained during the development of the reference scores in the following step.

The consensus reference labels of ASA-PS scores for the tuning and test datasets were developed by a consensus committee comprising three board-certified practicing anesthesiologists. The pre-anesthesia evaluation summaries were evaluated independently by two board-certified anesthesiologists. Any disagreements between the board-certified anesthesiologists were resolved via discussion or consulting with a third board-certified anesthesiologist. Five other board-certified anesthesiologists were excluded from the committee, and three anesthesiology residents were individually assigned the ASA-PS scores in the test dataset. These scores were used to compare the performance of the model with that of the individual ASA-PS providers with different levels of expertise. Thus, each record in the test dataset received one consensus reference label of ASA-PS score from the committee, five from the board-certified anesthesiologists, and three from the anesthesiology residents.

For evaluating GPT-4 performance^32^, we employed a few-shot prompting strategy, selecting one representative case from each ASA-PS class (1 through 5), resulting in a total of five in-context demonstrations. Each demonstration represented one ASA-PS class. The selection process for these examples involved initially randomly selecting ten cases per ASA-PS class. From these, our consensus committee then carefully chose the most representative case for ASA-PS class based on their clinical expertise. Furthermore, we consistently used the same five demonstrations in the few-shot prompting for each case to generate an ASA-PS prediction. Supplementary Table 2 presents the detailed prompts. The performance of GPT-4 in the test dataset was compared with that of the anesthesiology residents, board-certified anesthesiologists, and other language models.

### Model development

Baseline model architectures of ClinicalBigBird and BioClinicalBERT for ASA-PS classification were developed. These models used the pre-anesthesia evaluation as the input to output the ASA-PS score. Each model architecture underwent three learning stages in a sequential manner: (1) masked language modeling using the training dataset to learn the pre-anesthesia evaluation texts in a self-supervised manner, understanding the relationships between words; (2) supervised learning using the input and output training datasets; and (3) fine-tuning using the tuning dataset.

In the masked language modeling and supervised learning stages, we used a grid search approach to hyperparameter tuning, applying an 80:20 holdout method. For the fine-tuning stage, a 5-fold cross-validation approach was employed with a grid search to identify the best hyperparameters. The best parameters identified were then applied to train the models on the entire respective datasets. Early-stopping method based on validation loss was used to finalize the best model for each stage.

Supplementary Table 6 presents model training details with hyperparameters explored and their respective best values for each model. Throughout all learning stages, we used a cross-entropy loss function and the AdamW optimizer.

### Model evaluation

The AUROC, AUPRC, sensitivity, specificity, precision, recall, and F1-score were used to evaluate the performance of the models in the test set. Ten iterations were conducted for each pre-anesthesia evaluation summary to determine the probability distribution of the ASA-PS classes in GPT-4. To evaluate the average ASA-PS classification performances of the anesthesiology residents and the board-certified anesthesiologists, we first attempted to calculate the mode of the scorings given by the three anesthesiology residents and the five board-certified anesthesiologists, respectively, selecting the most frequently assigned ASA class. If all scores within each group were different, we used the mean value, rounded to the nearest whole number, as the final ASA-PS class. The AUROC and AUPRC were computed for each class and aggregated into macro and micro metrics according to the multi-class classification. To provide the real-world performance of the models, we also calculated the weighted average of the metrics using the prevalence of each ASA-PS class in our cohort. Confusion matrices for the predictions of each model and physician versus consensus reference labels were generated to identify the error distributions.

### Subgroup analysis

The performances of the models in the test set were compared and stratified according to the number of tokens as a part of the subgroup analysis. The test set was divided into two subgroups based on the length of each pre-anesthesia evaluation summary, with the median length of the test set used as a threshold.

### Model interpretability

The significance of each text affecting the ASA-PS classification and the reliance of the model on the interaction between texts was analyzed using the Shapley Additive exPlanations (SHAP) method. Examples of the importance of each word were plotted and overlaid on the original text. The SHAP force plots were used to illustrate how individual tokens within the pre-anesthesia evaluation summaries influence the model’s prediction for ASA-PS classification. Each force plot shows the contribution of each token to the model’s output probability. Tokens that push the prediction towards a higher probability of a specific class are colored red, while those that push it towards a lower probability are colored blue. The base value indicates the average prediction for the model, and the output value shows the specific prediction for the instance. The size of the arrows represents the magnitude of each token’s contribution, making it clear which tokens had the most significant impact on the final prediction.

### Statistical analysis

Data pre-processing, model development and validation, statistical testing, and visualization were performed using Python 3.10.0 (Python Software Foundation, Wilmington, DE, USA). The AUROCs of the different models were compared using the DeLong test. We measured two types of uncertainty in NLP models: aleatoric uncertainty, which could originate from the provided demonstrations, and epistemic uncertainty, which may be associated with the model’s configurations^33^. Specifically, we employ a Bayesian approach to estimate both uncertainties through Monte Carlo Dropout. For each input batch, we conduct multiple (1000 times) forward passes with active dropout, approximating Bayesian inference by sampling from the posterior distribution of model parameters. To capture aleatoric uncertainty, we introduce noise to the input data during each forward pass, reflecting inherent data variability. We aggregate the predicted probabilities from these passes to calculate the mean and variance for each class. Aleatoric uncertainty is quantified by the entropy of the mean predicted probabilities, while epistemic uncertainty is estimated by the mean variance of predictions. The consistency between the three anesthesiology residents and among the five anesthesiologists was evaluated using Fleiss’ kappa^34^. Kappa values of 0.01–0.20 were interpreted as ‘none to slight’, 0.21–0.40 as ‘fair’, 0.41–0.60 as ‘moderate’, 0.61–0.80 as ‘substantial’, and 0.81–1.00 as ‘almost perfect’ agreement^35^. The 95% CI for all evaluation metrics was computed using 1000 bootstrap iterations with 4000 bootstrap samples on the test set. A *p*-value <0.05 was considered statistically significant.

## Supplementary information

Supplemental material

## Data Availability

To the extent allowed by data sharing agreements and IRB protocols, the data from this manuscript will be shared upon written request.

## Code availability

The code used in this study can be accessed at: https://github.com/jipyeong-lee/ASA-PS-NLP-vs-Human-Physicians/.
