# Supplementary material for: Comparison of NLP machine learning models with human physicians for ASA Physical Status classification
Source: NPJ Digit Med. 2024 Sep 28;7:259. doi: 10.1038/s41746-024-01259-6 (PMC11439044; doi:10.1038/s41746-024-01259-6)
Supplement: Supplementary file 1 — Supplemental material [file 41746_2024_1259_MOESM1_ESM.pdf]

Supplementary Table 1. An example of translating a pre-anesthesia evaluation record written in a mixture of Korean and English using a proprietary translator

Before translation<br>(Written in a mixture of Korean and English)	After translation
* LLD->prone / EP(-) / EBL: mod /*Hb 13.0<br># L4-5-S1 foraminal stenosis @Sx: Rp to Rt L/E<br># HTN on ARB /@wBP 134/84-146/84 /@ECG: nsr<br># bMR abn @Sx: dizziness, headache, nausea, vomiting (발생 6 시간 후 호전) /@bMRI/A('23.2, o/s) no acute stroke in brain. poss small old infarction in Lt cerebellum. Lt prox M1 mild-mod stenosis. suspicious aneurysmal dilatation, Lt pCOM. fr(-) /@Sx: Sq: //NRc> stroke 는 배제 가능. periop stroke risk moderate 이상. 수술전후 충분한 hydration, mBP >65mmHg 유지, 갑작스러운 저혈압/고혈압 주의	* LLD->prone / EP(-) / EBL: mod /*Hb 13.0<br># L4-5-S1 foramal stenosis @Sx: Rp to Rt L/E<br># HTN on ARB /@wBP 134/84-146/84 /@ECG: nsr<br># bMR abn @Sx: dizziness, headache, nausea, vomiting (improved after 6 hours of occurrence) /@bMRI/A ('23.2, o/s) No acute stroke in brain. Possible small old infarctions in left cerebellum. Left proximity M1 mild-mod stenosis. Suspicious aneurysm dilatation, left pCOM. Fr (-) /@Sx: sq//nrc> Stroke is unlikely. Periop stroke risk moderate or higher. Adequate hydration before and after surgery, maintain mBP >65 mmHg, beware sudden hypotension/hypertension

This table demonstrates how the proprietary translator was used to convert pre-anesthesia evaluation records into English. LLD, left lateral decubitus; EP, epidural; EBL, estimated blood loss; mod, moderate; Hb, hemoglobin; Sx, symptoms; Rp, radiating pain; Rt, right; L/E, lower extremity; HTN, hypertension; ARB, angiotensin receptor blocker; wBP, ward blood pressure; ECG, electrocardiogram; nsr, normal sinus rhythm; bMR, brain magnetic resonance; abn, abnormal; bMRI/A, brain magnetic resonance imaging and angiography; o/s, onset; pCOM, posterior communicating artery; fr, formal reading; Sq, sequelae; NRc, neurology consultation; periop, perioperative; mBP, mean blood pressure.

Supplementary Table 2. Prompts for GPT-4, including instructions and guidelines for the ASA-PS classification system and one-shot for each ASA-PS class

(Instructions)
I would like you to assume the role of an anesthesiologist.
You should look at the patient preoperative evaluation summary and provide a correct ASA-PS score at the end.

(Guidelines)
Here are guidelines and examples for each ASA-PS score.
$$Guideline$$
ASA-PS 1: A normal healthy patient
Example: Healthy, non-smoking, no or minimal alcohol use
ASA-PS 2: A patient with mild systemic disease
Example: Mild diseases only without substantive functional limitations. Current smoker, social alcohol drinker, pregnancy, obesity (30<BMI<40), well-controlled DM/HTN, mild lung disease
ASA-PS 3: A patient with severe systemic disease
Example: Substantive functional limitations; One or more moderate to severe diseases. Poorly controlled DM or HTN, COPD, morbid obesity (BMI ≥40), active hepatitis, alcohol dependence or abuse, implanted pacemaker, moderate reduction of ejection fraction, ESRD undergoing regularly scheduled dialysis, history (>3 months) of MI, CVA, TIA, or CAD/stents.
ASA-PS 4: A patient with severe systemic disease that is a constant threat to life
Example: Recent (<3 months) MI, CVA, TIA or CAD/stents, ongoing cardiac ischemia or severe valve dysfunction, severe reduction of ejection fraction, shock, sepsis, DIC, ARD or ESRD not undergoing regularly scheduled dialysis
ASA-PS 5: A moribund patient who is not expected to survive without the operation
Example: Ruptured abdominal/thoracic aneurysm, massive trauma, intracranial bleed with mass effect, ischemic bowel in the face of significant cardiac pathology or multiple organ/system dysfunction
ASA-PS 6: A declared brain-dead patient whose organs are being removed for donor purposes

(One-shot for each ASA-PS class)
Here are some examples of patient preoperative evaluation summaries and the correct ASA-PS scores.

[Example 1]<br>$$Patient summary$$<br># Right ovarian cyst r/o teratoma (8.6 cm) @Hb: 13.6<br>#h/o thyroid nodule @TFT: WNL<br>* Before admission	$$ASA-PS score$$<br>1
[Example 2]<br>$$Patient summary$$<br>#Atypical Endometrial hyperplasia (endometrial thickness 1.3 cm, os) @Hb:14.0<br>#Allergic rhinitis on antihistamine @URI Sx (-), sore throat (+)	$$ASA-PS score$$<br>2
[Example 3]<br>$$Patient summary$$<br># Rectosigmoid colon cancer (5.6 cm, cT4bN+)<br>/@abdCT('22.3.24): a 5.6 cm rectosigmoid jxn ca. located 13.7 cm above from the AV c uterus and ovary direct invasion, 6.5 cm and 3.8 cm multilocular cystic lesions, both ovaries(r/o bilateral kruckenburg tumors > primary ovarian cystic neoplasms)<br># @PFT 1.64(91%)/1.74(68%)=94 (mild restrictive) /@CPA nl /@cCT('23.3.25) no evidence of intrathoracic meta. /*Sx (-) /@SpO_2 95-97<br>*BMI 32.05(157.4 cm/79.4 kg)	$$ASA-PS score$$<br>3
[Example 4]<br>$$Patient summary$$<br>*ICU arranged<br>#Ovarian ca- s/p open LSO, omentectomy, appendectomy('18, os), s/p CTx (paclitaxel-carboplatin#6, ~'18.10), s/p pelvicoscopic RSO, terminal ileum serosa repair('20.7, G/A s event), s/p CTx(Padexol-carboplatin-bevacizumab#6, ~'20.11., ~'21.10.13 bevacizumab+), s/p explo lapa('21.10, G/A, TAH, omentectomy, B)paracolic gutter peritonectomy, rectal mass excision, R)diaphragmatic excision done), s/p CTx(Doxil-carbo#6, ~'22.4)/@APCT('23.2): Multifocal seeding lesions with malignant ascites, subdiaphragmatic, perisplenic, perihepatic, mesentery, paracolic gutter, pelvic cavity. Cardiophrenic LN metastases/@PET('23.3): Probable multiple peritoneal seeding. No definite evidence of extraabdominal meta/@CBC: 3800-12.9-194K<br>@PT/INR/aPTT: 10.7/0.98/40.2 s▲<br>*Flomoxef allergy (+)	$$ASA-PS score$$<br>4
[Example 5]<br>$$Patient summary$$<br>* ICU pt. (covid+)<br>* GCS 4/E/4<br>* BMI 11.3 (150 cm/25.4 kg)	$$ASA-PS score$$<br>5

* V/S: BP 92/63-116/63 HR 170-180, SpO_2 (FiO_2): 96-97%, BT 38.2
* on NEPI 0.05 mcg/kg/min
*@Hb/INR/PLT/aPTT 8.4▼/1.95▲/69k▼/62.2>> RBC /FFP t/f state
* Rt. subclavian C-line, Rt. hand pph line, L-tube in situ, Rt. PCD in situ
* intubated state (plain 6.5 cuffed, @17cm) on SIMV-PC mode (FiO_2 0.4, set PC 12, set Fx 22, set PEEP 8, act VT 121-150) /* pleural effusion(+) @A'(FiO_2 0.4): 7.37-45-39-26 (71.7%) /*sl. yellow secretion(+)
# Sigmoid colon perforation /@Sx: hematemesis /@Alb 2.4
# Covid 19 pneumoniae (4/1-) c pleural effusion /@cCT('23.4.6):Moderate amount of bilateral pleural effusion.(Sub)segmental atelectasis in both lungs. Minimal pericardial effusion. Scoliosis. No significant LNE in mediastinum. No remarkable findings in the covered upper abdomen.
# Rett syndrome /@bCT('23.4.6):Suspicious low density lesion with poor GM-WM differentiation on precontrast image - not definite on contrast image--> pseudolesion, more likely
# @ECG: S. tachy(196), nonsp intraventricular conduction delay
# @E' 148-4.4-117

(Final instruction)
Based on $$Guideline$$ and these examples, provide a step-by-step evaluation on the new patient summary at $$Explanation$$, and a correct $$ASA-PS score$$ at the end.
Always give one answer, even when the answer is ambiguous.

The prompt includes instructions for the ASA-PS classification system and examples for one-shot learning per ASA-PS class to ensure accurate predictions. ASA-PS, American Society of Anesthesiologists Physical Status; GPT-4, Generative Pretrained Transformer-4; BMI, Body Mass Index; DM, Diabetes Mellitus; HTN, Hypertension; COPD, Chronic Obstructive Pulmonary Disease; ESRD, End-Stage Renal Disease; MI, Myocardial Infarction; CVA, Cerebrovascular Accident; TIA, Transient Ischemic Attack; CAD, Coronary Artery Disease; DIC, Disseminated Intravascular Coagulation; ARD, Acute Respiratory Distress; r/o, rule out; Hb, Hemoglobin; h/o, history of; TFT, Thyroid Function Test; WNL, Within Normal Limits; URI, Upper Respiratory Infection; Sx, Symptoms; cT4bN+, clinical stage T4b, Node positive; @abdCT, Abdominal Computed Tomography; jxn, junction; AV, Anal Verge; @PFT, Pulmonary Function Test; @CPA, Costophrenic Angle; @cCT, Chest Computed Tomography; @SpO_2, Peripheral Oxygen Saturation; ICU, Intensive Care Unit; ca, cancer; s/p, status post; LSO, Left Salpingo-Oophorectomy; CTx, Chemotherapy; RSO, Right Salpingo-Oophorectomy; G/A, General Anesthesia; TAH, Total Abdominal Hysterectomy; APCT, Abdominopelvic Computed Tomography; LN, Lymph Node; PET, Positron Emission Tomography; meta, metastasis; CBC, Complete Blood Count; PT, Prothrombin Time; INR, International Normalized Ratio; aPTT, activated Partial Thromboplastin Time; pt, patient; covid+, COVID-19 positive; GCS, Glasgow Coma Scale; E, Eye response in GCS; V/S, Vital Signs; BP, Blood Pressure; HR, Heart Rate; FiO_2, Fraction of Inspired Oxygen; BT, Body Temperature; NEPI, Norepinephrine; PLT, Platelets; RBC, Red Blood Cells; FFP, Fresh Frozen Plasma; t/f, transfusion; C-line, Central line; pph, peripheral; L-tube, Levin tube; PCD, Percutaneous Catheter Drainage; SIMV-PC, Synchronized Intermittent Mandatory Ventilation-Pressure Control; PC, Pressure Control; Fx, Frequency; PEEP, Positive End-Expiratory Pressure; VT, Tidal Volume; Alb, Albumin; cCT, Chest Computed Tomography; LNE, Lymph Node Enlargement; bCT, Brain Computed Tomography; GM-WM, Grey Matter-White Matter; ECG, Electrocardiogram; tachy, tachycardia; E', electrolyte.

Supplementary Table 3. ASA-PS classification performances of the individual board-certified anesthesiologist and anesthesiology resident

	Sensitivity (Recall)	Specificity	Precision	F1-score
**Anesthesiologist 1**				
Weighted-average	0.752 (0.737–0.766)	0.846 (0.837–0.854)	0.638 (0.622–0.655)	0.668 (0.652–0.684)
Macro-average	0.729 (0.715–0.741)	0.889 (0.884–0.893)	0.672 (0.658–0.686)	0.675 (0.661–0.690)
Micro-average	0.670 (0.656–0.684)	0.670 (0.656–0.684)	0.670 (0.656–0.684)	0.670 (0.656–0.684)
**Anesthesiologist 2**				
Weighted-average	0.731 (0.717–0.744)	0.843 (0.835–0.851)	0.626 (0.610–0.642)	0.633 (0.615–0.650)
Macro-average	0.729 (0.715–0.741)	0.888 (0.883–0.892)	0.689 (0.674–0.703)	0.679 (0.664–0.694)
Micro-average	0.663 (0.648–0.677)	0.663 (0.648–0.677)	0.663 (0.648–0.677)	0.663 (0.648–0.677)
**Anesthesiologist 3**				
Weighted-average	0.760 (0.747–0.773)	0.857 (0.849–0.865)	0.655 (0.639–0.671)	0.671 (0.655–0.688)
Macro-average	0.786 (0.776–0.797)	0.902 (0.898–0.907)	0.724 (0.711–0.738)	0.726 (0.713–0.740)
Micro-average	0.707 (0.693–0.721)	0.707 (0.693–0.721)	0.707 (0.693–0.721)	0.707 (0.693–0.721)
**Anesthesiologist 4**				
Weighted-average	0.809 (0.797–0.820)	0.851 (0.843–0.859)	0.667 (0.651–0.682)	0.709 (0.693–0.724)
Macro-average	0.782 (0.770–0.793)	0.905 (0.900–0.909)	0.739 (0.725–0.753)	0.734 (0.720–0.747)
Micro-average	0.719 (0.706–0.732)	0.719 (0.706–0.732)	0.719 (0.706–0.732)	0.719 (0.706–0.732)
**Anesthesiologist 5**				
Weighted-average	0.743 (0.729–0.757)	0.810 (0.801–0.820)	0.598 (0.582–0.615)	0.640 (0.623–0.657)
Macro-average	0.723 (0.711–0.736)	0.879 (0.874–0.884)	0.680 (0.667–0.695)	0.669 (0.655–0.683)
Micro-average	0.648 (0.633–0.662)	0.648 (0.633–0.662)	0.648 (0.633–0.662)	0.648 (0.633–0.662)
**Resident 1**				
Weighted-average	0.678 (0.660–0.695)	0.835 (0.825–0.843)	0.780 (0.766–0.793)	0.674 (0.654–0.694)
Macro-average	0.625 (0.608–0.641)	0.883 (0.878–0.888)	0.789 (0.773–0.804)	0.664 (0.646–0.680)
Micro-average	0.700 (0.685–0.713)	0.700 (0.685–0.713)	0.700 (0.685–0.713)	0.700 (0.685–0.713)
**Resident 2**				
Weighted-average	0.607 (0.593–0.622)	0.729 (0.720–0.738)	0.616 (0.591–0.641)	0.533 (0.514–0.553)
Macro-average	0.508 (0.492–0.523)	0.840 (0.835–0.845)	0.677 (0.654–0.697)	0.527 (0.509–0.546)
Micro-average	0.587 (0.573–0.602)	0.587 (0.573–0.602)	0.587 (0.573–0.602)	0.587 (0.573–0.602)
**Resident 3**				
Weighted-average	0.676 (0.658–0.693)	0.789 (0.780–0.798)	0.702 (0.683–0.722)	0.639 (0.618–0.659)
Macro-average	0.625 (0.610–0.641)	0.861 (0.856–0.866)	0.688 (0.670–0.705)	0.611 (0.594–0.628)

Micro-average	0.628 (0.612–0.644)	0.628 (0.612–0.644)	0.628 (0.612–0.644)	0.628 (0.612–0.644)

This table presents the performance metrics (Sensitivity, Specificity, Precision, and F1-score) of individual board-certified anesthesiologists and anesthesiology residents when classifying ASA-PS scores. Data are presented as means with 95% confidence intervals. ASA-PS, American Society of Anesthesiologists Physical Status.

Supplementary Table 4. Inter-rater reliability among different raters in each dataset

Study	Raters	Number of cases	Fleiss' kappa	95% confidence interval
Mak et al. 2022 [1]	Board-certified anesthesiologists (n=96)	10	0.34	0.34-0.35
Ours – Tuning set	Board-certified anesthesiologists (n=5)	426	0.811	0.801-0.822
Ours – Test set	Board-certified anesthesiologists (n=5)	460	0.743	0.731-0.754
Ours – Test set	Anesthesiology residents (n=3)	460	0.480	0.463-0.498
Ours – Test set	GPT-4 (n=10)	460	0.612	0.601-0.623

Inter-rater reliability was measured by Fleiss' kappa among different groups of raters, including board-certified anesthesiologists, anesthesiology residents, and GPT-4.

Supplementary Table 5. **Aleatoric and epistemic uncertainties of NLP models**

	Predicted ASA-PS							
	ASA-PS I		ASA-PS II		ASA-PS III		ASA-PS IV-V	
	AU	EU	AU	EU	AU	EU	AU	EU
ClinicalBigBird (random)								
Consensus reference labels								
ASA-PS I	0.623 (0.606-0.640)	0.002 (0.002-0.002)	0.803 (0.789, 0.818)	0.004 (0.003-0.004)				
ASA-PS II	0.682 (0.660-0.701)	0.002 (0.002-0.002)	0.725 (0.717-0.734)	0.003 (0.003-0.003)	0.809 (0.780-0.839)	0.006 (0.004-0.008)		
ASA-PS III			0.766 (0.753-0.779)	0.003 (0.003-0.003)	0.770 (0.762-0.778)	0.005 (0.005-0.005)	0.647 (0.609-0.682)	0.008 (0.006-0.009)
ASA-PS IV-V			0.897 (0.840-0.956)	0.004 (0.003-0.004)	0.752 (0.728-0.774)	0.007 (0.006-0.008)	0.557 (0.529-0.584)	0.004 (0.004-0.005)
BioClinicalBERT (random)								
Consensus reference labels								
ASA-PS I	0.706 (0.676-0.736)	0.005 (0.004-0.006)	1.009 (0.987-1.030)	0.005 (0.005, 0.007)	1.363 (1.360-1.366)	0.032 (0.032-0.033)	1.193 (1.172-1.219)	0.046 (0.045-0.046)
ASA-PS II	0.851 (0.810-0.891)	0.006 (0.005-0.007)	0.935 (0.924-0.947)	0.005 (0.004-0.005)	0.960 (0.935-0.986)	0.007 (0.006-0.008)	1.128 (1.064-1.188)	0.022 (0.020-0.023)
ASA-PS III			1.032 (1.013-1.052)	0.005 (0.004-0.006)	0.971 (0.957-0.984)	0.006 (0.006-0.007)	0.976 (0.924-1.024	0.011 (0.010-0.012)
ASA-PS IV-V			1.257 (1.238-1.273)	0.007 (0.006-0.008)	0.947 (0.924-0.972)	0.006 (0.006, 0.007)	0.777 (0.736, 0.815)	0.006 (0.006-0.007)
ClinicalBigBird (class-stratified)								
Consensus reference labels								
ASA-PS I	0.662 (0.644-0.678)	0.003 (0.003, 0.003)	0.749 (0.739-0.761)	0.003 (0.003-0.004)	0.919 (0.879-0.954)	0.011 (0.009-0.013)		
ASA-PS II	0.685 (0.655-0.715)	0.002 (0.002-0.003)	0.711 (0.703-0.719)	0.003 (0.003-0.003)	0.824 (0.809-0.840)	0.006 (0.005-0.007)	0.587 (0.534-0.633)	0.010 (0.008-0.011)
ASA-PS III			0.773 (0.759-0.786)	0.004 (0.004-0.004)	0.777 (0.771-0.783)	0.006 (0.006-0.006)	0.604 (0.578-0.630)	0.007 (0.006-0.008)
ASA-PS IV-V			0.861 (0.848-0.876)	0.006 (0.005-0.007)	0.764 (0.738-0.790)	0.008 (0.006-0.009)	0.477 (0.457-0.498)	0.003 (0.003-0.003)
BioClinicalBERT (class-stratified)								
Consensus reference labels								

ASA-PS I	0.651 (0.636-0.667)	0.003 (0.003-0.003)	0.801 (0.790-0.811)	0.004 (0.004-0.005)				
ASA-PS II	0.708 (0.699-0.718)	0.003 (0.003-0.004)	0.728 (0.720-0.736)	0.004 (0.004-0.004)	0.794 (0.773-0.817)	0.008 (0.007-0.009)	0.748 (0.699-0.787)	0.023 (0.020-0.026)
ASA-PS III			0.789 (0.771-0.808)	0.006 (0.006-0.007)	0.753 (0.745-0.762)	0.012 (0.011-0.012)	0.635 (0.608-0.660)	0.016 (0.014-0.017)
ASA-PS IV-V			0.988 (0.972-1.008)	0.016 (0.014-0.018)	0.717 (0.692-0.744)	0.014 (0.012-0.016)	0.467 (0.444-0.491)	0.007 (0.006-0.008)

The '(random)' and '(class-stratified)' terms indicate different sampling methods used in the uncertainty estimation process. When making input batches for uncertainty estimation, random sampling selected cases without considering the distribution of ASA-PS classes across batches, while class-stratified sampling ensured equal distribution of ASA-PS classes across batches. Data are presented as means with 95% confidence intervals. NLP, natural language processing; AU, aleatoric uncertainty; EU, epistemic uncertainty; ASA-PS, American Society of Anesthesiologists Physical Status.

Supplementary Table 6. Details of the hyperparameters explored and their respective best values for each stage.

	Search space of hyperparameters	Best hyperparameters for ClinicalBigBird	Best hyperparameters for BioClinicalBERT
**1st learning stage: masked language modeling using the training dataset**			
Batch size	32, 64, 128, 256	256	256
Learning rate	5e-7	5e-7	5e-7
Learning rate scheduler type	Linear, Cosine	Cosine	Cosine
Masking ratio	0.15	0.15	0.15
**2nd learning stage: supervised learning using the training dataset**			
Batch size	32, 64, 128, 256	256	256
Learning rate	1e-7	1e-7	1e-7
Learning rate scheduler type	Linear, Cosine	Cosine	Cosine
Label smoothing factor	0.1, 0.2	0.2	0.2
**3rd learning stage: fine-tuning using the tuning dataset**			
Batch size	2, 4, 8, 16	4	4
Learning rate	5e-7	5e-7	5e-7
Learning rate scheduler type	Linear, Cosine	Linear	Linear
Label smoothing factor	0.1, 0.2	0.2	0.2

This table presents the search space for hyperparameters and the best-performing configurations used in the final models.

Supplementary References

[1] Mak, P. H. K., Campbell, R. C. H., & Irwin, M. G. The ASA physical status classification: inter-observer consistency. *Anaesth Intensive Care* **30**, 633-640 (2002).
